# Quantitative proteomic analysis of GnRH agonist treated GBM cell line LN229 revealed regulatory proteins inhibiting cancer cell proliferation

**DOI:** 10.1186/s12885-022-09218-8

**Published:** 2022-02-02

**Authors:** Priyanka H. Tripathi, Javed Akhtar, Jyoti Arora, Ravindra Kumar Saran, Neetu Mishra, Ravindra Varma Polisetty, Ravi Sirdeshmukh, Poonam Gautam

**Affiliations:** 1 Laboratory of Molecular Oncology, ICMR- National Institute of Pathology, Safdarjung Hospital Campus, New Delhi, 110029, India; 2 Jamia Hamdard- Institute of Molecular Medicine, Jamia Hamdard, New Delhi, 110062, India; 3 Govind Ballabh Pant Institute of Postgraduate Medical Education and Research (GIPMER), New Delhi, 110002 India; 4 Symbiosis International (Deemed University), Pune, 412115 India; 5 Department of Biochemistry, Sri Venkateswara College, University of Delhi, New Delhi, 110021, India; 6 Institute of Bioinformatics, International Tech Park, Bangalore, 560066, India; 7 Manipal Academy of Higher Education (MAHE), Manipal, 576104, India

**Keywords:** Glioblastoma, Gonadotropin-Releasing Hormone receptor, iTRAQ, Proteome

## Abstract

**Background:**

Gonadotropin-releasing hormone (GnRH) receptor, a rhodopsin-like G-protein coupled receptor (GPCR) family member involved in GnRH signaling, is reported to be expressed in several tumors including glioblastoma multiforme (GBM), one of the most malignant and aggressive forms of primary brain tumors**.** However, the molecular targets associated with GnRH receptor are not well studied in GBM or in other cancers. The present study aims at investigating the effect of GnRH agonist (Gosarelin acetate) on cell proliferation and associated signaling pathways in GBM cell line, LN229.

**Methods:**

LN229 cells were treated with different concentrations of GnRH agonist (10^−10^ M to 10^−5^ M) and the effect on cell proliferation was analyzed by cell count method. Further, total protein was extracted from control and GnRH agonist treated cells (with maximum reduction in cell proliferation) followed by trypsin digestion, labeling with iTRAQ reagents and LC-MS/MS analysis to identify differentially expressed proteins. Bioinformatic analysis was performed for annotation of proteins for the associated molecular function, altered pathways and network analysis using STRING database.

**Results:**

The treatment with different concentrations of GnRH agonist showed a reduction in cell proliferation with a maximum reduction of 48.2% observed at 10^−6^ M. Quantitative proteomic analysis after GnRH agonist treatment (10^−6^ M) led to the identification of a total of 29 differentially expressed proteins with 1.3-fold change (23 upregulated, such as, kininogen-1 (KNG1), alpha-2-HS-glycoprotein (AHSG), alpha-fetoprotein (AFP), and 6 downregulated, such as integrator complex subunit 11 (CPSF3L), protein FRG1 (FRG1). Some of them are known [KNG1, AHSG, AFP] while others such as inter-alpha-trypsin inhibitor heavy chain H2 (ITIH2), ITIH4, and LIM domain-containing protein 1 (LIMD1) are novel to GnRH signaling pathway. Protein-protein interaction analysis showed a direct interaction of KNG1, a hub molecule, with GnRH, GnRH receptor, EGFR and other interactors including ITIH2, ITIH4 and AHSG. Overexpression of KNG1 after GnRH agonist treatment was validated using Western blot analysis, while a significant inhibition of EGFR was observed after GnRH agonist treatment.

**Conclusions:**

The study suggests a possible link of GnRH signaling with EGFR signaling pathways likely via KNG1. KNG1 inhibitors may be investigated independently or in combination with GnRH agonist for therapeutic applications.

**Supplementary Information:**

The online version contains supplementary material available at 10.1186/s12885-022-09218-8.

## Background

Glioblastoma multiforme (GBM) is among the most aggressive brain tumor with a poor mean survival period of 12–14 months [1]. Chemoresistance and recurrence is common among these tumors and therefore poses a serious challenge to treatment management [1]. It is important to identify novel drugs/drug targets for improved treatment of this cancer.

Gonadotropin-releasing hormone (GnRH), agonists have been shown to have direct anti-proliferative effects on various cancer cell lines from prostate, breast, ovary, and endometrium [2]. Functional studies with GnRH receptor knockdown showed an inhibitory effect on cell invasion, migration and cell proliferation in various cancer cell lines [3–8]. Though, targeted studies show its link with growth factor receptors and integrins [2], the mechanism of action of GnRH and GnRH receptor (GnRHR) in cancer cells is not fully understood.

Expression of GnRH and GnRH receptor have been reported in GBM tissue samples and cell lines. Marelli *et al* showed that treatment of GBM cell lines (U87MG and U373) with GnRH agonists (Zoladex) results in significant reduction (42.5%) in cell proliferation. They also showed that GnRH agonist is able to inhibit GBM cell proliferation by reducing cAMP levels, induced by forskolin *in vitro*, suggesting that GnRH receptors may be coupled to Gαi-cAMP intracellular signaling pathway [9]. In another study, Jaszberenyi *et al* showed that treatment of U87MG xenograft nude mice with GnRH analog, AN-152**,** almost completely abolished tumor progression *in vivo* (76% reduction in tumor growth) and showed that AN-152 elicited remarkable anti-proliferation activity and apoptosis *in vitro*. Further, they analyzed 84 cancer associated genes and showed nuclear factor κB (NF-κB), platelet derived growth factor (PDGF), matrix metallopeptidase 9 (MMP-9), urokinase plasminogen activator (uPA), melanoma cell adhesion molecule (MCAM), metastasis associated 1 family, member 2 (MTA2) to be significantly altered after AN-152 treatment [10].

Earlier, we analyzed differentially regulated kinases in GBM, from high-throughput proteomic and transcriptomic datasets using tumor tissue, which revealed the association of these kinases to ‘GnRH signaling pathway’ [11]. Its plausible cross-connectivity with epithelial growth factor receptor (EGFR), Wnt, calcium, and focal adhesion kinase signaling pathways was shown in GBM. The GnRH pathway was curated with extensive literature analysis that led to a comprehensive update of the pathway. In the present study, we analyzed proteomic changes upon treatment with GnRH agonist to understand molecular processes associated with GnRH signaling.

## Methods

### GBM cell line

LN229, a commonly used glioblastoma cell line, was employed to study the effect of GnRH agonist treatment and identify differentially expressed proteins using quantitative proteomics. The cells were cultured in DMEM media (Thermo Fisher Scientific, USA) supplemented with 10% fetal bovine serum (FBS) (Thermo Fisher Scientific, USA), 1% penicillin/streptomycin (Thermo Fisher Scientific, USA). Cells were passaged at ~80% confluency.

### Chemicals

The GnRH agonist Goserelin acetate [Glp-His-Trp-Ser-Tyr-Ser(tBu)-Leu-Arg-Pro-azaGly-NH2 or D-Ser (tBu)AzaGly-GnRH] (Sigma, USA) was used for the experiment.

### RT-PCR analysis

The GBM cells, (LN229) were plated in 25 cm^2^ flask in DMEM medium supplemented with 10% FBS (complete media) and cultured in 5% CO_2_ at 37 °C. The cells were allowed to attach and start growing till 70–80% confluency. RNA was isolated using TRIzol Reagent (Life technologies, USA) according to the protocol from the manufacturer. The quantity and quality were checked using NanoDrop 2000 (Thermo Scientific, USA) and 1.5% agarose gel electrophoresis respectively. First cDNA synthesis was carried out using 1 μg of isolated RNA and High-Capacity cDNA Reverse Transcription Kit (Invitrogen, Life Technologies). Later, RT-PCR was performed to analyze the expression of GnRH receptor using cDNA template, gene specific primers (Forward primer 5’AGGCTTGAAGCTCTGTTGTCCTG-3′ and Reverse primer 5′-CATGAAGGCTGGGGCATACA-3′) and Taq DNA polymerase kit (Invitrogen, Life Technologies) as per the manufacturer’s protocol. For amplification of GnRHR cDNA, PCR was performed for 35 cycles (30 s denaturation at 95 °C, 30 s primer annealing at 60 °C and 45 s primer extension at 72 °C). The PCR product was separated on 1.5% agarose gel stained with ethidium bromide.

### Western Blot analysis

LN229 cells were collected at 70–80% confluency for protein extraction. Cells were scrapped out and resuspended in modified RIPA buffer [25 mM Tris-Cl, pH 7.6 + 150 mM NaCl +2% (3-[(3-cholamidopropyl) dimethylammonio]-1-propanesulfonate (CHAPS)] with 1% PMSF protease inhibitor followed by sonication. Protein concentration was determined using Bradford assay. A total of 15 μg protein was resolved by 10% SDS-PAGE and stained with Coomassie brilliant blue R250 to study the protein profile. Western blot analysis was performed to study the expression of GnRH receptor. Briefly, the protein was resolved by SDS-PAGE and electro-transferred to a PVDF membrane (Millipore, Bedford, MA), blocked with 5% (v/v) skimmed milk in TBST (150 mM NaCl, 20 mM Tris, 0.1% Tween 20, pH 7.4) for 2 h at room temperature, followed by incubation with primary antibodies (GnRH receptor monoclonal antibody, dilution 1:1000- ThermoFisher Scientific) diluted with 2.5% skimmed milk in TBST at room temperature for 2 h. After extensive wash with TBST, the membrane was incubated with horseradish peroxidase-conjugated secondary antibody (anti-mouse IgG HRP conjugated; Thermo, USA; dilution 1:20,000) diluted with 2.5% skimmed milk in TBST for 90 min at room temperature. The membrane was developed using Immobilon Western chemiluminescent horseradish peroxidase substrate (Millipore). Densitometric analysis of the specific band showing reactivity was done to get relative expression of GnRH receptor in LN229.

### Clinical samples

A total of 23 Clinical samples (10 GBM cases, 9 epilepsy cases and 4 pituitary adenoma) (FFPE tissue, retrospective cases) used were obtained from Govind Ballabh Pant Institute of Postgraduate Medical Education and Research (GIPMER), New Delhi after approval of the ICMR-National Institute of Pathology- Institutional Ethics Committee, New Delhi (NIP-IEC).

### Immunohistochemistry analysis

The expression level of GnRH receptor protein was studied in cases (GBM cases, *n* = 10), non-tumor controls (epilepsy cases, *n* = 9) and positive control (pituitary adenoma, *n* = 4) by immunohistochemistry analysis as described earlier by Polisetty *et al* [12]. In brief, after deparaffinization and rehydration of formalin-fixed paraffin-embedded (FFPE) tissue sections, antigen retrieval was performed by immersing the slide in antigen retrieval buffer (10 mM sodium citrate, 0.05% Tween 20, pH 6.0) at 95 °C for 5 min. Endogenous peroxidases were blocked with hydrogen peroxide, and nonspecific binding was blocked with 2% fetal calf serum in Tris-buffered saline with 0.1% Triton X-100 (TBST, pH 7.6). Sections were then incubated for 1 h at RT with primary antibody against GnRH receptor (dilution 1:100) (Thermo, USA) followed by peroxidase-labelled polymer conjugate to anti-rabbit or anti-mouse immunoglobulins compatible with the primary antibody, for 10 min and were developed with diaminobenzidine (DAB) system (Thermo, USA). Sections were counter stained with the Mayer’s hematoxylin, dehydrated and images were taken using light microscope. The staining distribution and staining intensity across the section was observed under the microscope. Scoring criteria were based on both staining intensities and distributions [13]. The staining intensity of cancer cells scored as 0, 1+, 2+/3+ indicating negative, low, and strong staining respectively. The distribution of staining of cancer cells was scored as 0 (< 10% of cells staining), 1+ (10- < 25% of cell staining), 2+ (25- < 50% of cells staining) and 3+ (≥50% of cells staining).

### Effect of GnRH agonist on cell proliferation using Cell Counting method

Cells were seeded at a density of 8000 cells/T25 Flask in DMEM medium. Cells were allowed to attach and start growing for 3 days. The seeding media was then replaced by experimental media containing GnRH agonist and the control flasks were replenished with DMEM media (without GnRH agonist). Cells were treated for 7 days with GnRH agonist (10^−10^ M- 10^−5^ M concentration) and medium was changed every two days. At the end of the treatment media was removed followed by washing with 1x PBS. Cells were trypsinized and resuspended in fresh medium. Cells were then stained with Trypan Blue (0.2%) for 10 s and cell counting was performed using Neubauer counting chamber. Based on cell counting, the percentage reduction in cell proliferation between control and GnRH agonist treated cells was calculated. The experiment was performed in triplicates.

### Quantitative proteomics analysis

The cells treated with GnRH agonist, at a concentration of 10^−6^ M, with a maximum reduction in cell proliferation, were further used to perform quantitative proteomic analysis to understand the downstream signaling pathways associated with GnRH signaling in GBM. GBM cells (Control and GnRH agonist treated) were resuspended in RIPA buffer with protease inhibitor and then sonicated to lyse the cells. Protein concentration was determined using Bradford assay. The experiment was performed twice. Proteins were reduced, alkylated and digested with trypsin followed by labelling with different iTRAQ reagents (control- 114, 115 and GnRH agonist treated- 116, 117) according to the manufacturer’s instructions (iTRAQ Reagents Multiplex kit; Applied Biosystems). The labeled samples were pooled, vacuum-dried and subjected to strong cation exchange (SCX) fractionation (*n* = 8 fractions) as described earlier [14]. The samples were desalted and lyophilized followed by mass spectrometric analysis (nano-LC MS/MS analysis) of each fraction.

#### LC-MS/MS analysis

Nanoflow electrospray ionization tandem mass spectrometric analysis was carried out using QExactive plus (Thermo Scientific, Bremen, Germany) interfaced with UltiMate™ 3000 RSLCnano System as described earlier by Priya *et al* [15]. Briefly, the peptides from each SCX fraction were enriched using a C18 trap column (75 μm × 2 cm) at a flow rate of 3 μl/min and fractionated on an analytical column (75 μm × 50 cm) at a flow rate of 300 nl/min using a linear gradient of 8–35% acetonitrile (ACN) over 85 min. Mass spectrometric analysis was performed in a data dependent manner using the Orbitrap mass analyzer at a mass resolution of 70,000 at m/z 200. For each MS cycle, 10 topmost intense precursor ions were selected and subjected to MS/MS fragmentation and detected at a mass resolution of 35,000 at m/z 200. The fragmentation was carried out using higher-energy collision dissociation (HCD) mode. Normalized collision energy (CE) of 30% was used to obtain the release of reporter ions from all peptides detected in the full scan. The ions selected for fragmentation were excluded for the next 30 s. The automatic gain control for full FT MS and FT MS/MS was set to 3e6 ions and 1e5 ions respectively with a maximum time of accumulation of 50 msec for MS and 75 msec for MS/MS. The lock mass with 10 ppm error window option was enabled for accurate mass measurements.

#### Data analysis

Protein identification, quantification and annotations of differentially expressed proteins were carried out as follows. The MS/MS data was analyzed using Proteome Discoverer (Thermo Fisher Scientific, version 1.4) with Mascot and Sequest HT search engine nodes using the NCBI RefSeq database (release 81). Search parameters included trypsin as the enzyme with 1 missed cleavage allowed; precursor and fragment mass tolerance were set to 10 ppm and 0.1 Da, respectively; Methionine oxidation and deamidation of asparagines and glutamine amino acids was set as a dynamic modification while methylthio modification at cysteine and iTRAQ modification at N-terminus of the peptide and lysines were set as static modifications. The peptide and protein information was extracted using high peptide confidence and top one peptide rank filters. The labeling efficiency was determined to be >99%. The iTRAQ data was normalized and the normalized values are provided in Supplementary Table S1. The variation in total intensity among different reporter tags was <3% for an average of the control and agonist treated sample. The FDR was calculated using percolator node in proteome discoverer 1.4. High confidence peptide identifications were obtained by setting a target FDR threshold of 1% at the peptide level. Relative quantitation of proteins was carried out based on the intensities of reporter ions released during MS/MS fragmentation of peptides. The average relative intensities of the two reporter ions for each of the unique peptide identifiers for a protein were used to determine the relative quantity of a protein and percentage variability [15]. Appropriate filters at the peptides/peptide spectral matches (PSMs) level and then at the protein level were applied to derive the quantification values as described earlier by Polisetty *et al* [16]

- First, only Peptide/PSMs that are unique for a protein were selected for fold change calculation.
- We selected a protein subset with 1.2-fold change cut-off. Next, peptide/PSMs with higher than 30% variability between the replicate label measurements (i.e., 114 and 115 for control) or (i.e., 116 and 117 for test i.e. GnRH agonist treated) were removed from the entire set of raw files.
- We then calculated four independent ratios (116/114, 117/114, 116/115 and 117/115 derived from internal technical replicates) for all PSMs and % CV value was determined for each of the PSMs included in the dataset. Similarly, we also calculated % CV values across all PSMs with each of the four ratios, contributing to each of the significant proteins in the dataset. For more than 95% of the proteins, the % CV calculated as above were found to be below 40%.
- Proteins with 1.3-fold change and above in GnRH agonist treated cells were considered significant and used for further analysis. The % CV values are shown in Supplementary Table S1 (see results).
- The student t-test was performed using the intensity value of PSMs from the two experimental replicates for a particular protein from control and agonist treated cells to calculate the p-value. Proteins with 1.3-fold change in expression level and p-value <0.05 was considered for identification of differentially expressed proteins.

### Bioinformatic analysis

Annotation for molecular functions, cellular localization, biological processes, pathways and protein-protein interaction analysis of the identified differentially expressed proteins was carried out using Search Tool for the Retrieval of Interacting Genes/Proteins (STRING) database version 11.0 (https://string-db.org/) [17].

### EGFR and KNG1 expression in GnRH agonist treated cells using Western blot analysis

Western blot analysis was performed to study the expression of epidermal growth factor receptor (EGFR) and KNG1 in control and GnRH agonist treated cells. Initially, a total of 15 μg protein from control and GnRH agonist treated cells was resolved by 10% SDS-PAGE followed by visualization of proteins by staining with Coomassie R250 Brilliant Blue. Densitometric analysis was performed to normalize the protein load in both the samples. The normalized protein amount from control and GnRH agonist treated cell lysate was used for Western blot analysis. Briefly, an equal protein amount (15 μg) was loaded on to the SDS-PAGE gel followed by electro transfer of proteins to a PVDF membrane (Millipore, Bedford, MA). The membrane was blocked with 5% skimmed milk in TBST (150 mM NaCl, 20 mM Tris, 0.1% Tween 20, pH 7.4) for 2 h at room temperature, followed by incubation with primary antibody (EGFR monoclonal antibody-Thermo; dilution 1:2000) and KNG1 (dilution 1:5000) diluted with 2.5% skimmed milk in TBST at room temperature for 2 h. After washing with TBST, the membrane was incubated with horseradish peroxidase-conjugated secondary antibody (anti-rabbit IgG HRP conjugated- Thermo; dilution 1:30,000) diluted with 2.5% skimmed milk in TBST for 90 min at room temperature. The membrane was developed using Immobilon Western chemiluminescent horseradish peroxidase substrate (Millipore). Densitometric analysis of the specific band showing reactivity was carried out for relative expression of EGFR in GnRH agonist treated cells. The experiment was performed thrice.

## Results

**Fig. 1:**
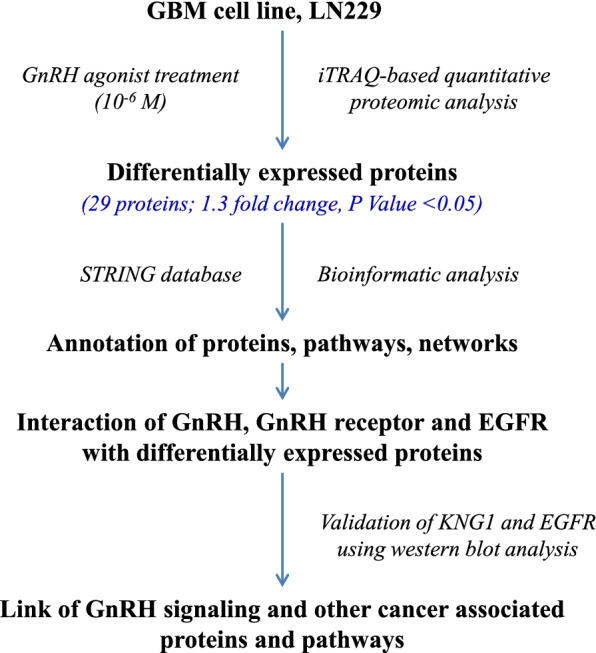
Overall workflow of the study

The present study analyzed the effect of GnRH agonist on cell proliferation in GBM cell line, LN229 by iTRAQ-based quantitative proteomic analysis. The differentially expressed proteins were annotated for their cellular components, molecular functions, biological processes, pathways and networks associated with these proteins using STRING database. The effect of GnRH agonist on the expression of KNG1 and a well-known oncogene, EGFR, was analyzed using Western blot. The overall workflow of the study is shown in Fig. 1.

### Expression of GnRH receptor in GBM cell line and tumor tissue samples

**Fig. 2:**
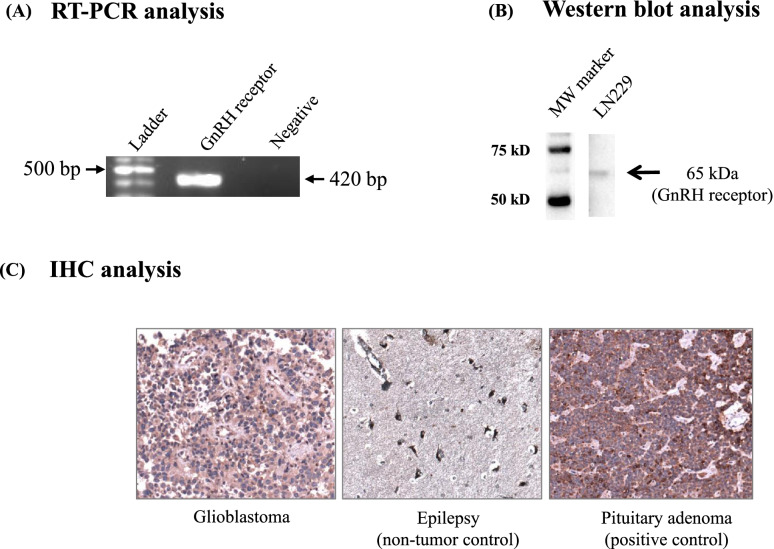
Expression of GnRH receptor in GBM cell line and tumor tissue samples. **A** RT-PCR analysis confirming expression of GnRH receptor (420 bp) (**B**) Western blot showing expression of GnRH receptor at 65 kDa, in LN229 cell line (**C**) Representative immunohistochemistry (IHC) image showing expression of GnRH receptor in GBM tumor tissue and non-tumor (epilepsy) tissue samples. IHC analysis showed the expression of GnRH receptor in 4 out of 10 GBM tissue samples. Epilepsy cases, used as non-tumor control, showed negative expression of GnRH receptor in astrocytic cells. Pituitary adenoma was used as a positive control. (Magnification- 10×). Full-length blot images are presented in Supplementary Fig. 1A and B. Full-length IHC images with a scale bar and magnification are presented in Supplementary Fig. 1E

We observed GnRH receptor expression in GBM cell line, LN229, both at the transcript and protein level. RT-PCR analysis showed a PCR product of 420 bp confirming the expression of GnRH receptor in GBM cell line **(**Fig. 2A, Supplementary Fig. S1A). Western blot analysis performed using LN229 cell lysate showed the expression of GnRH receptor at ~63 kD (Fig. 2B, Supplementary Fig. S1B).

We also analyzed the expression of GnRH receptor in GBM tumor tissue using immunohistochemistry (IHC) analysis using FFPE tissue sections and found the ‘strong’ expression in four out of ten (40%) GBM cases while all the non-tumor controls (epilepsy cases) showed ‘negative’ expression (Supplementary Table S2). The representative IHC images are shown in Fig. 2C**.**

### Effect of GnRH agonist treatment on cell proliferation in GBM cell line

**Fig. 3:**
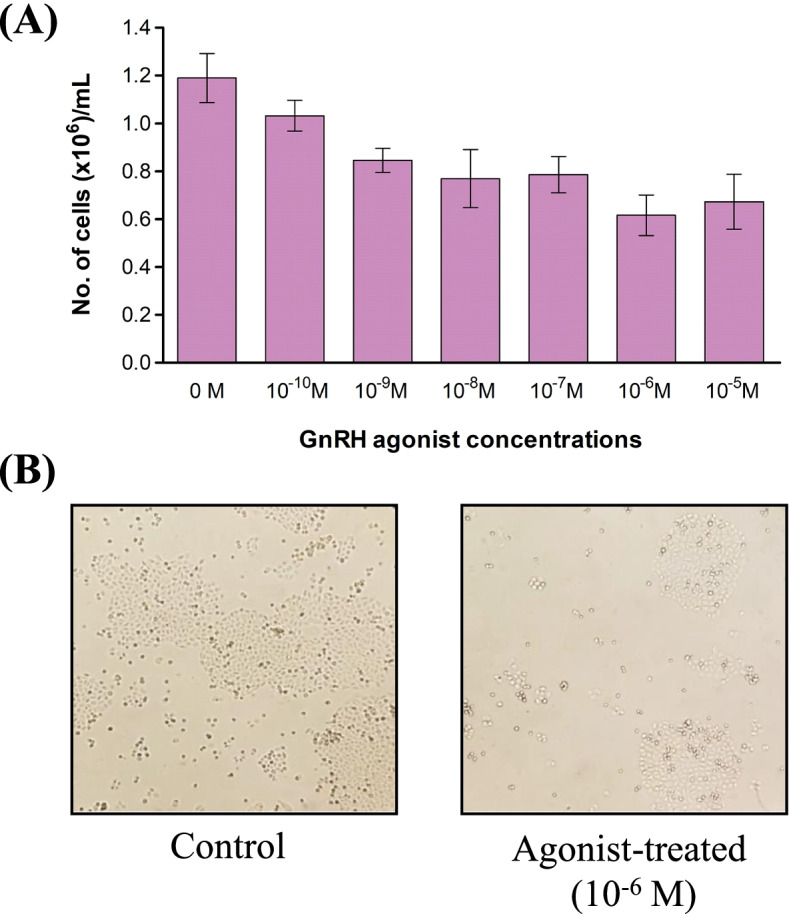
Effect of GnRH agonist treatment on cell proliferation in GBM cell line, LN229. Treatment of GBM cell line, LN229, with GnRH agonist showed (**A**) a maximum reduction (48.3%) in cell proliferation at 10^−6^ M concentration as determined by cell count using a hemocytometer. The error bars represent the standard error of mean (**B**) LN229 cells with and without GnRH agonist treatment, 10 × Magnification

The effect of GnRH agonist treatment on cell proliferation in LN229 cells was analyzed using cell counting method using Trypan blue cell viability assay. We observed 13.2–48.2% reduction in cell proliferation at 10^−10^ M- 10^−5^ M concentration with a maximum reduction in cell proliferation (i.e. 48.2%) was observed at 10^−6^ M concentration (Fig. 3A and B). Earlier, Marelli *et al* [9] found maximum reduction in cell proliferation at a similar concentration of Zoladex (or goserelin) in two GBM cell lines, U87MG and U373. Overall, the effective GnRH agonist concentration was similar for the three cell lines, U87MG, U373 and LN229. We planned to analyze the proteins and pathways differentially expressed by GnRH agonist at this concentration using quantitative proteomic analysis.

### iTRAQ based quantitative proteomics analysis

Quantitative proteomic analysis of LN229 cells after GnRH agonist treatment led to the identification of a total of 3180 proteins (1988 proteins were identified by ≥2 unique peptides), of these 29 proteins were identified with ≥1.3 fold change in expression level and p-value <0.05 after GnRH agonist treatment (Supplementary Table S1). Among these, ~50% of the proteins were with 1.5 fold change and above. A total of 23 proteins are upregulated [e.g. kininogen-1 (KNG1), alpha-2-HS-glycoprotein (AHSG), alpha-fetoprotein (AFP), inter-alpha-trypsin inhibitor heavy chain H2 (ITIH2), inter-alpha-trypsin inhibitor heavy chain H4 isoform 2 (ITIH4), pancreatic lipase-related protein 3 (PNLIPRP3)] and 6 were found to be downregulated [e.g. integrator complex subunit 11 (CPSF3L), protein FRG1 (FRG1), calcium and integrin-binding protein 1 (CIB1), LIM domain-containing protein 1 (LIMD1)] (Table 1).

**Table 1:** List of 29 proteins differentially expressed proteins after GnRH agonist treatment

S. No.	Gene Symbol	Gene ID	Protein	Protein Fold change	***P***-Value
1	ITIH2	3698	inter-alpha-trypsin inhibitor heavy chain H2	1.709	0.0000
2	ITIH4	3700	inter-alpha-trypsin inhibitor heavy chain H4 isoform 2	1.523	0.0007
3	SIK3	23,387	PREDICTED: serine/threonine-protein kinase SIK3 isoform X9	1.258	0.0007
4	AFP	174	alpha-fetoprotein	1.572	0.0011
5	C3	718	complement C3	1.369	0.0017
6	AHSG	197	alpha-2-HS-glycoprotein	1.668	0.0017
7	PNLIPRP3	119,548	PREDICTED: pancreatic lipase-related protein 3 isoform X1	1.883	0.0019
8	BRD9	65,980	PREDICTED: bromodomain-containing protein 9 isoform X2	1.274	0.0021
9	KNG1	3827	kininogen-1 isoform 3	1.405	0.0032
10	C7	730	complement component C7	1.474	0.0042
11	APOA1	335	apolipoprotein A-I isoform 1	1.526	0.0043
12	CFAP100	348,807	PREDICTED: cilia- and flagella-associated protein 100 isoform X9	1.506	0.0046
13	GC	2638	PREDICTED: vitamin D-binding protein isoform X1	1.494	0.0050
14	TRMT10C	54,931	mitochondrial ribonuclease P protein 1	1.283	0.0050
15	ATP7B	540	PREDICTED: copper-transporting ATPase 2 isoform X11	1.664	0.0067
16	LTF	4057	lactotransferrin isoform 2	1.646	0.0068
17	LIMD1	8994	LIM domain-containing protein 1	0.361	0.0075
18	CWF19L1	55,280	CWF19-like protein 1 isoform 4	0.742	0.0079
19	EXOC4	60,412	PREDICTED: exocyst complex component 4 isoform X3	1.533	0.0086
20	APOH	350	beta-2-glycoprotein 1	1.508	0.0087
21	TMOD2	29,767	tropomodulin-2 isoform b	1.474	0.0111
22	CIB1	10,519	calcium and integrin-binding protein 1 isoform b	0.614	0.0135
23	CPSF3L	54,973	integrator complex subunit 11 isoform 5	0.713	0.0153
24	STK39	27,347	PREDICTED: STE20/SPS1-related proline-alanine-rich protein kinase isoform X5	1.266	0.0174
25	PPARD	5467	peroxisome proliferator-activated receptor delta isoform 4	1.812	0.0198
26	FRG1	2483	protein FRG1	0.696	0.0221
27	GSTCD	79,807	glutathione S-transferase C-terminal domain-containing protein isoform 2	1.790	0.0303
28	NKTR	4820	PREDICTED: NK-tumor recognition protein isoform X3	1.459	0.0372
29	HTRA1	5654	serine protease HTRA1	0.690	0.0423

### Bioinformatic analysis

The proteins differentially expressed after GnRH agonist treatment were annotated for cellular components, molecular functions, biological processes, pathways and networks using STRING database. Gene Ontology annotations showed vesicle lumen, secretory granule lumen, extracellular region, endoplasmic reticulum lumen, extracellular space as top ‘cellular components’. The top ‘biological processes’ include regulation of response to external stimulus, regulation of inflammatory response, regulation of defense response, regulation of response to stress, negative regulation of endopeptidase activity. Endopeptidase inhibitor activity, enzyme inhibitor activity, enzyme regulator activity, molecular function regulator and cysteine-type endopeptidase inhibitor activity were among the top altered ‘molecular functions’ **(**Table 2A**)**.

**Table 2:** Annotation of proteins deregulated after GnRH agonist treatment for (A) their molecular functions, biological processes and cellular components and (B) Reactome Pathways using STRING database

**(A) Molecular functions, biological processes and cellular components**				
**Biological Process (GO)**				
**S. No.**	**GO-term**	**Description**	**Count in gene set**	**False discovery rate**
1	GO:0032101	Regulation of response to external stimulus	11 of 955	0.00033
2	GO:0050727	Regulation of inflammatory response	7 of 338	0.00085
3	GO:0031347	Regulation of defense response	9 of 676	0.00085
4	GO:0080134	Regulation of response to stress	11 of 1299	0.001
5	GO:0010951	Negative regulation of endopeptidase activity	6 of 242	0.001
**Molecular Function (GO)**				
**S. No.**	**GO-term**	**Description**	**Count in gene set**	**False discovery rate**
1	GO:0004866	Endopeptidase inhibitor activity	6 of 169	0.0000413
2	GO:0004857	Enzyme inhibitor activity	8 of 388	0.0000413
3	GO:0030234	Enzyme regulator activity	9 of 1016	0.001
4	GO:0098772	Molecular function regulator	11 of 1793	0.0027
5	GO:0004869	Cysteine-type endopeptidase inhibitor activity	3 of 57	0.0037
**Cellular Component (GO)**				
**S. No.**	**GO-term**	**Description**	**Count in gene set**	**False discovery rate**
1	GO:0031983	Vesicle lumen	8 of 341	0.0000152
2	GO:0034774	Secretory granule lumen	7 of 323	0.000095
3	GO:0005576	Extracellular region	15 of 2505	0.00015
4	GO:0005788	Endoplasmic reticulum lumen	6 of 299	0.00039
5	GO:0005615	Extracellular space	9 of 1134	0.0023
**(B) PATHWAYS**				
**S. No.**	**Pathway**	**Description**	**Count in gene set**	**False discovery rate**
1	HSA-381426	Regulation of Insulin-like Growth Factor (IGF) transport and uptake by Insulin-like Growth Factor Binding Proteins (IGFBPs)	2 of 38	0.031
2	HSA-109582	Hemostasis	5 of 591	0.031

Pathway analysis using STRING database (Reactome pathway) showed two pathways to be significantly altered including regulation of insulin-like growth Factor (IGF) transport and uptake by Insulin-like Growth Factor Binding Proteins (IGFBPs) and hemostasis (Table 2B**).**

**Fig. 4:**
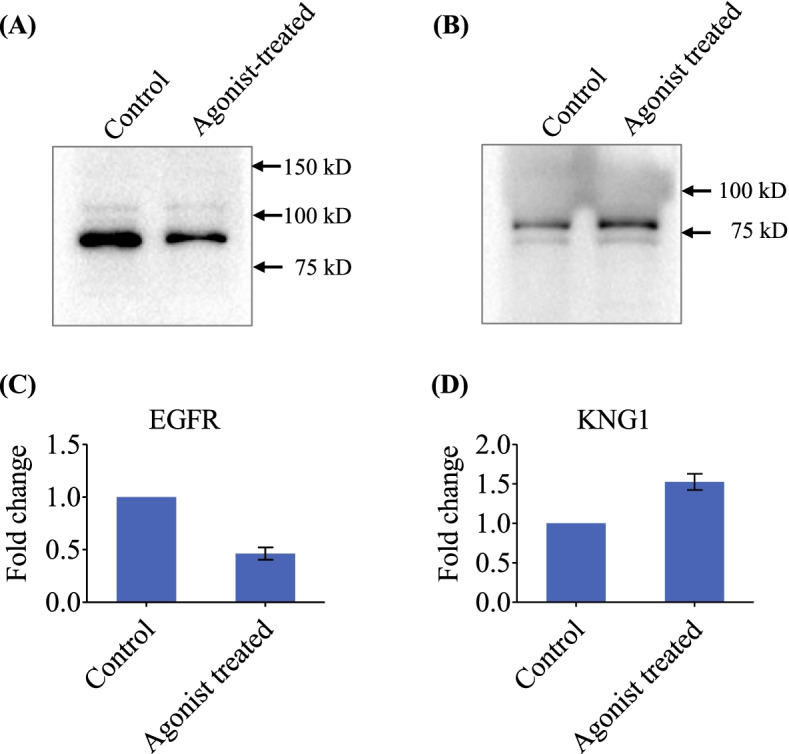
EGFR and KNG1 expression in control and GnRH agonist treated LN229 cells using Western blot analysis. The densitometric analysis of the Western blot shows a significant reduction in EGFR (2.2 fold) (A) and overexpression of KNG1 (1.5 fold) (B) after GnRH agonist treatment in comparison to untreated cells. For Western blot analysis, a total of 15 μg protein from LN229 cell lysate (Control and GnRH agonist treated group) was resolved by SDS-PAGE and electrotransferred onto PVDF membrane. The membrane was incubated with primary antibody against EGFR (dilution 1:2000) and KNG1 (dilution 1:5000) followed by incubation with anti-rabbit secondary antibody (dilution 1:30,000). The blots were developed using ECL reagent, image was acquired using Chemidoc MP (Bio-Rad) followed by densitometric analysis. The bar diagram shows the expression level of EGFR (C) and KNG1 (D) in control and agonist treated cell lysate. Error bars represent the standard deviation of mean. Full-length blot images are presented in Supplementary Fig. 1C and D

**Fig. 5:**
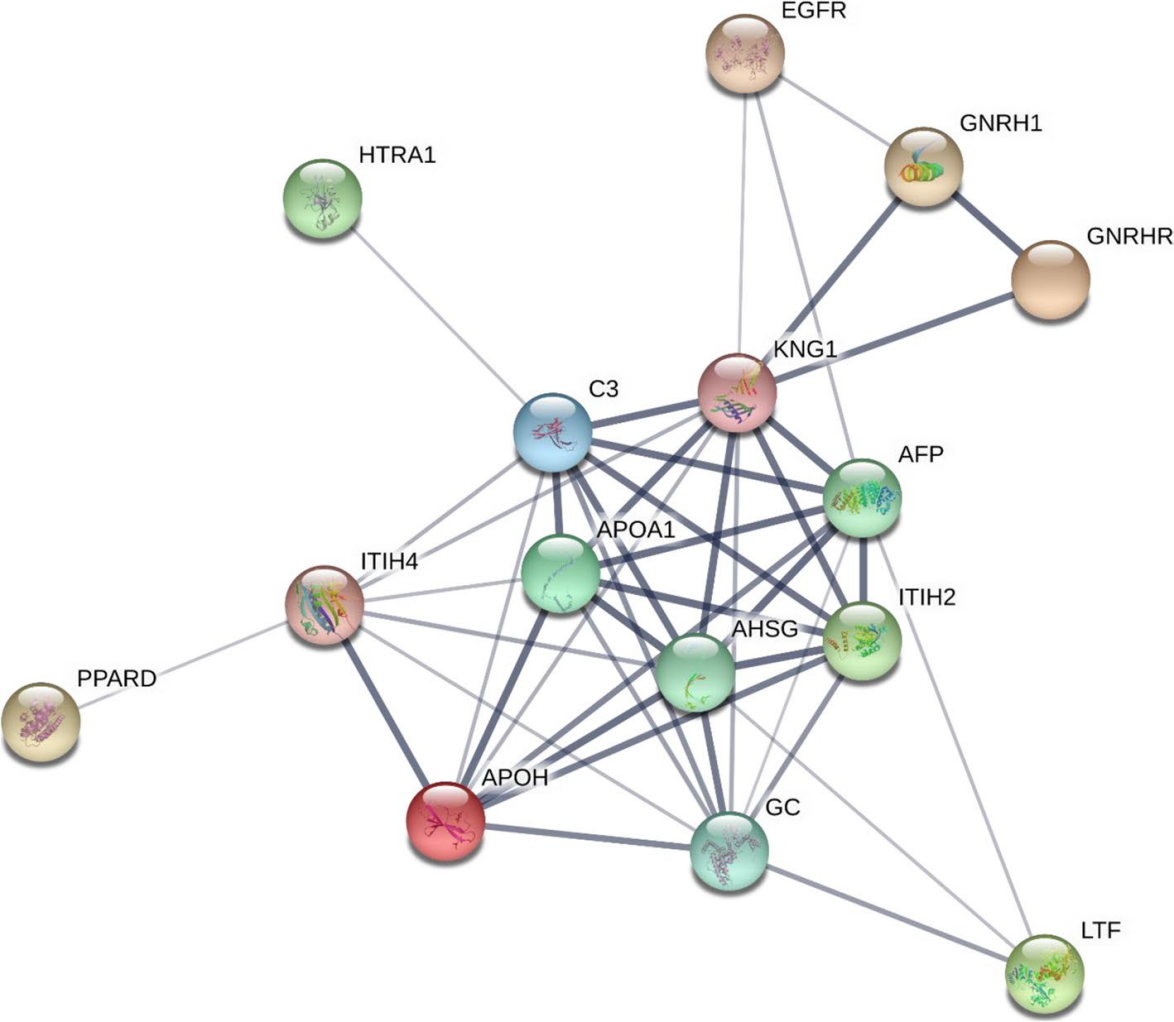
Protein-protein interaction analysis of differentially expressed proteins after GnRH agonist treatment. GnRH, GnRH receptor and EGFR, not detected in the proteomics data, were included for the protein-protein interaction analysis that showed KNG1 to be interacting directly with GnRH, GnRH receptor, EGFR and 8 other proteins. *Line thickness indicates the strength of data support*

We performed protein-protein interaction (PPi) analysis, using differentially expressed protein set to understand their relevance to GnRH signaling. Our earlier effort on updating GnRH pathway [17], revealed a possible cross-connectivity between GnRH and EGFR signaling. We further validated the expression of EGFR by Western blot analysis and found 2.2 fold downregulation after GnRH agonist treatment (Fig. 4A, C, Supplementary Fig. S1C). Therefore, for the PPi network to find out proteins interacting with GnRH and GnRHR, we used the dataset of 29 differentially expressed proteins observed after treatment with GnRH agonist, as well as GnRH, GnRHR and EGFR, although not detected in the proteomics analysis presumably due to their low abundance. We found 15 proteins showing one or more direct or indirect interactions. KNG1, AHSG, AFP, complement 3 (C3) were among the top four hub molecules. We observed KNG1 to be interacting with GnRH, GnRHR, EGFR and 8 other proteins including AFP, AHSG, C3, APOA1, ITIH2, GC, ITIH4 and APOH (Fig. 5). KNG1 expression was validated by Western blot analysis and observed 1.5 fold overexpression after GnRH agonist treatment (Fig. 4B, D, Supplementary Fig. S1D).

## Discussion

The role of GnRH signaling in cell proliferation has been earlier established in various cancers. There are several efforts, to understand the molecular processes associated with GnRH signaling in cancer. In the present study, we applied quantitative proteomic analysis to study the effect of GnRH agonist (goserelin acetate) in GBM cell line, LN229, and to understand the molecular processes associated with GnRH signaling. Literature search for the 29 differentially expressed proteins identified in this study showed 6 of them to have an association with GnRH signaling pathway including KNG1, AHSG, AFP, FRG1, lactotransferrin isoform 2 (LTF), peroxisome proliferator-activated receptor delta isoform 4 (PPARD), while the remaining 23 proteins are novel to GnRH signaling in GBM or other cancers, which includes ITIH2 and ITIH4. Protein-protein interaction network analysis of these proteins showed KNG1 to be a direct interactor of GnRH, GnRHR, EGFR and 8 other interactors, including ITIH2, AHSG and AFP (Fig. 5).

KNG1 is a cysteine proteinase inhibitor and is reported to inhibit cell proliferation and angiogenesis. Xu *et al* reported a significantly low expression of KNG1 at transcript level after analysis of TCGA dataset including 169 tumor samples from GBM patients and 5 normal samples while a high KNG1 expression was reported to be associated with increased survival in glioma patients**.** Further, the study revealed that overexpression of KNG1 promotes apoptosis and G1 phase cell cycle arrest which demonstrates its role in inhibiting tumor growth in glioma cells [18]. A cleaved domain 5 of high molecular weight kininogen (HK) is reported to bind to urokinase-type plasminogen activation receptor (uPAR) with high affinity and inhibit EGFR phosphorylation leading to significant reduction of cell migration and invasion in human prostate cancer cells [19]. GnRH agonist treatment, using both *in vitro* and *in vivo* studies, in various cancers showed a significant downregulation of EGFR [2]. In the present study, quantitative proteomic analysis showed higher expression of KNG1 (1.4 fold change) in GnRH agonist treated cells. Independently, we observed overexpression of KNG1 (1.5 fold) and downregulation of EGFR (2.2 fold) in response to the treatment of GnRH agonist by Western blot analysis (Fig. 4). These results suggest a possible association of GnRH and EGFR signaling via KNG1 in GBM.

STRING analysis showed KNG1 further interacts with 8 other proteins including AFP, AHSG, C3, APOA1, ITIH2, GC, ITIH4 and APOH (Fig. 5). The literature search showed co-expression of the interacting proteins in cancer or other clinical conditions. KNG1 and ITIH4 were reported to be significantly downregulated in ovarian cancer [20]. Post-translational modification in KNG1, AHSG and downregulation of ITIH2 has been reported in colorectal cancer [21]. Analysis of primary human brain tumors showed significantly higher levels of ITIH2 in normal brain and low-grade tumors compared with high-grade gliomas, indicating an inverse correlation with malignancy [22]. Stable overexpression of ITIH2 in U251 glioma cells leads to strong inhibition of cancer cell invasion together with significant inhibition of cell proliferation and promotion of cell-cell adhesion. Further, overexpression of ITIH2 led to downregulation of phospho-AKT suggesting its link to phosphatidylinositol 3-kinase/Akt signaling cascade, therefore, restoring the ITIH2 supply exogenously could be useful for therapeutic applications [23]. Inhibition of PI3k/AKT functional activity has been observed after overexpression of KNG1 in glioma cells [18]. The KNG1 interaction with other proteins may be validated and explored further for therapeutic applications.

## Conclusions

The present study analyzed the molecular processes associated with GnRH signaling and revealed GnRH and GnRHR interaction with KNG1, a hub molecule, which might be involved in regulating cell proliferation in GBM through modulation of EGFR pathway.

## Supplementary Information

**Additional file 1.**

## Data Availability

All data generated or analyzed for this study is included in the main article and supplementary information files and is also available with the corresponding author.

## Abbreviations

GBM: Glioblastoma multiforme
GnRH: Gonadotropin-releasing hormone
RT-PCR: Reverse transcription polymerase chain reaction
DMEM: Dulbecco’s Modified Eagle Medium
FBS: Fetal Bovine Serum
cDNA: Complementary DNA
PVDF: Polyvinylidene fluoride
PMSF: Phenylmethylsulfonyl fluoride
FFPE: Formalin-fixed paraffin-embedded
SCX: Strong cation exchange
iTRAQ: Isobaric tag for relative and absolute quantitation
FDR: False discovery rate
CV: Coefficient of variation
TCGA: The Cancer Genome Atlas
cAMP: Cyclic adenosine monophosphate
