# Supplementary material for: Quantitative proteomic analysis of GnRH agonist treated GBM cell line LN229 revealed regulatory proteins inhibiting cancer cell proliferation
Source: BMC Cancer. 2022 Feb 2;22:133. doi: 10.1186/s12885-022-09218-8 (PMC8812247; doi:10.1186/s12885-022-09218-8)
Supplement: Supplementary file 1 — Additional file 1. [file 12885_2022_9218_MOESM1_ESM.zip › Supplementary Fig. S1 Full length images.pptx]

## Slide 1
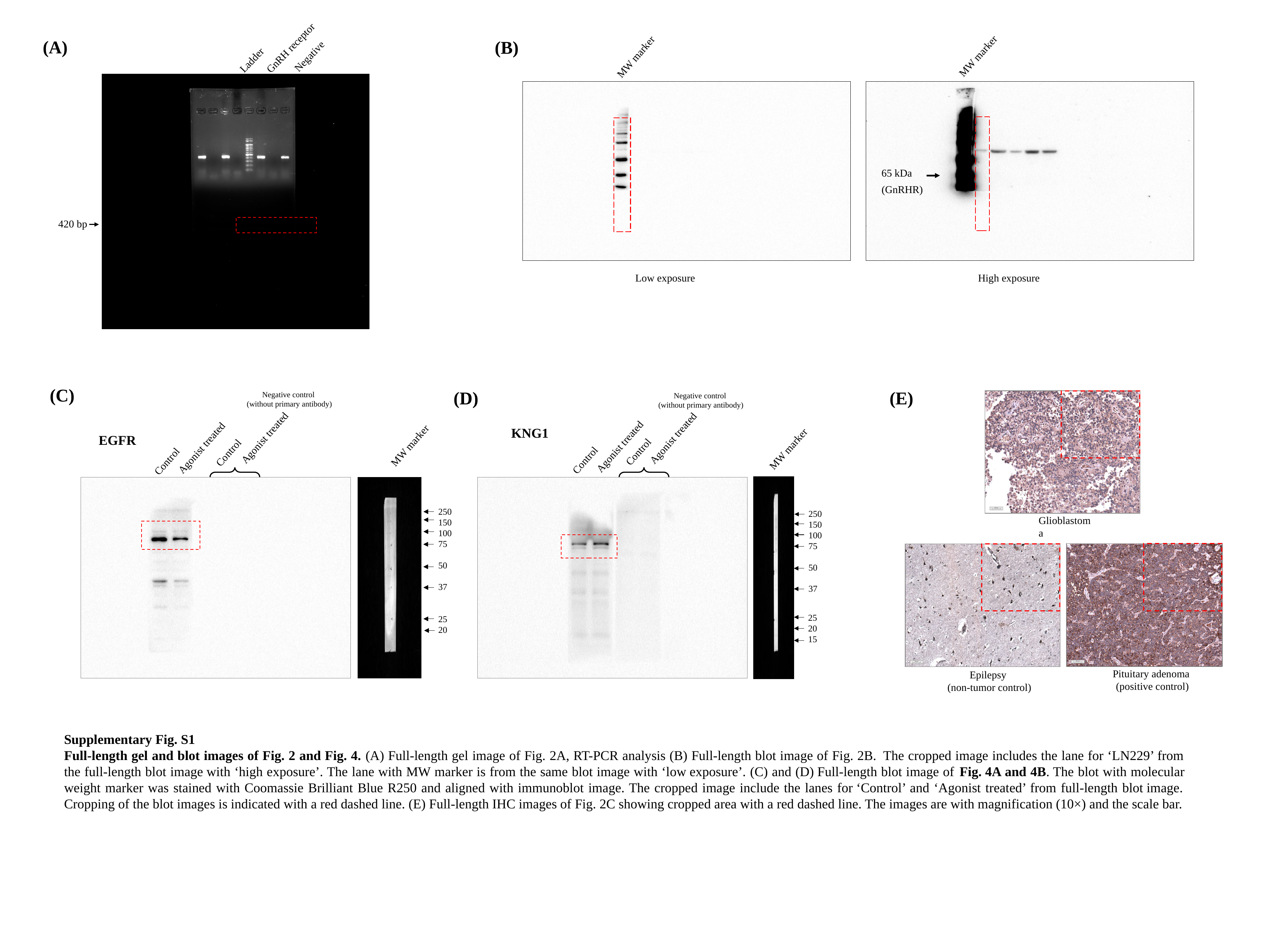

GnRH receptor
Negative
Ladder
420 bp
MW marker
MW marker
65 kDa
(GnRHR)
Low exposure
High exposure
(B)
(A)
(C)
Negative control
(without primary antibody)
Agonist treated
Control
Agonist treated
Control
(D)
Agonist treated
MW marker
Control
(E)
Negative control
(without primary antibody)
KNG1
Agonist treated
EGFR
MW marker
Control
250
150
100
75
50
37
25
20
250
150
100
75
50
37
Glioblastoma
25
20
15
Pituitary adenoma
(positive control)
Epilepsy
(non-tumor control)
Supplementary Fig. S1
Full-length gel and blot images of Fig. 2 and Fig. 4. (A) Full-length gel image of Fig. 2A, RT-PCR analysis (B) Full-length blot image of Fig. 2B.  The cropped image includes the lane for ‘LN229’ from the full-length blot image with ‘high exposure’. The lane with MW marker is from the same blot image with ‘low exposure’. (C) and (D) Full-length blot image of Fig. 4A and 4B. The blot with molecular weight marker was stained with Coomassie Brilliant Blue R250 and aligned with immunoblot image. The cropped image include the lanes for ‘Control’ and ‘Agonist treated’ from full-length blot image. Cropping of the blot images is indicated with a red dashed line. (E) Full-length IHC images of Fig. 2C showing cropped area with a red dashed line. The images are with magnification (10×) and the scale bar.
