# Supplementary material for: Quantitative proteomic analysis of GnRH agonist treated GBM cell line LN229 revealed regulatory proteins inhibiting cancer cell proliferation
Source: BMC Cancer. 2022 Feb 2;22:133. doi: 10.1186/s12885-022-09218-8 (PMC8812247; doi:10.1186/s12885-022-09218-8)
Supplement: Supplementary file 1 — Additional file 1. [file 12885_2022_9218_MOESM1_ESM.zip › Supplementary Table S2.doc]

**Supplementary Table S2**

**Protein expression of GnRH receptor in GBM tissue samples using immunohistochemistry analysis.** IHC was performed using FFPE tissue section from 10 GBM cases, 09 epilepsy cases (normal control) and 04 pituitary adenoma (positive control). We found ‘strong’ expression in four out of ten (40%) GBM cases while all the non-tumor controls (epilepsy cases) showed ‘negative’ expression. Scoring criteria used were based on both staining intensities and distributions. Scoring criteria were based on both staining intensities and distributions. The staining intensity of cancer cells scored as 0, 1+, 2+/3+ indicating negative, weak, and strong staining respectively. The distribution of staining of cancer cells was scored as 0 (≤10% of cells staining), 1+ (>10-25% of cell staining), 2+ (>25-50% of cells staining) and 3+ (>50% of cells staining). Representative IHC image of tumor tissue from GBM and normal temporal cortex of epilepsy cases (as control) is shown in **Figure 2C.**

| **Subjects**  **(No. of subjects)** | **Staining intensity** | | | |
| --- | --- | --- | --- | --- |
|  | **0** | **1+** | **2+** | **3+** |
| **GBM cases (10)** | 6 | 2 | 2 | 0 |
| **Epilepsy (09)** | 9 | 0 | 0 | 0 |
| **Pituitary adenoma (04)** | 0 | 0 | 0 | 4 |
